# Supplementary material for: Di(isononyl) cyclohexane-1,2-dicarboxylate (DINCH) alters transcriptional profiles, lipid metabolism and behavior in zebrafish larvae
Source: Heliyon. 2021 Sep 8;7(9):e07951. doi: 10.1016/j.heliyon.2021.e07951 (PMC8441171; doi:10.1016/j.heliyon.2021.e07951)
Supplement: Supplementary file 1 — Supplementary Table [file mmc1.docx]

**Primers**

Table S1. Primes used in this study.
